# Thermodynamic Analysis of the Solubility of Isoniazid in (PEG 200 + Water) Cosolvent Mixtures from 278.15 K to 318.15 K

**DOI:** 10.3390/ijms231710190

**Published:** 2022-09-05

**Authors:** Daniela Baracaldo-Santamaría, Carlos Alberto Calderon-Ospina, Claudia Patricia Ortiz, Rossember Edén Cardenas-Torres, Fleming Martinez, Daniel Ricardo Delgado

**Affiliations:** 1 Pharmacology Unit, Department of Biomedical Sciences, School of Medicine and Health Sciences, Universidad del Rosario, Bogotá 111221, Colombia; 2 GENIUROS Research Group, Center for Research in Genetics and Genomics (CIGGUR), School of Medicine and Health Sciences, Universidad del Rosario, Bogotá 111221, Colombia; 3 Programa de Administración en Seguridad y Salud en el Trabajo, Grupo de Investigación en Seguridad y Salud en el Trabajo, Corporación Universitaria Minuto de Dios-UNIMINUTO, Neiva 410001, Colombia; 4 Grupo de Fisicoquímica y Análisis Matemático, Facultad de Ciencias y Humanidades, Fundación Universidad de América, Bogotá 111221, Colombia; 5 Grupo de Investigaciones Farmacéutico-Fisicoquímicas, Departamento de Farmacia, Facultad de Ciencias, Universidad Nacional de Colombia, Sede Bogotá, Carrera 30 No. 45-03, Bogotá 111221, Colombia; 6 Programa de Ingeniería Civil, Grupo de Investigación de Ingenierías UCC-Neiva, Facultad de Ingeniería, Universidad Cooperativa de Colombia, Sede Neiva, Neiva 410001, Colombia

**Keywords:** isoniazid, solubility, cosolvent, thermodynamics, PEG 200, water

## Abstract

The solubility of drugs in cosolvent systems of pharmaceutical interest is of great importance for understanding and optimizing a large number of processes. Here, we report the solubility of isoniazid in nine (PEG 200 + water) cosolvent mixtures at nine temperatures (278.15, 283.15, 288.15, 293.15, 298.15, 303.15, 308.15, and 318.15 K) determined by UV–vis spectrophotometry. From the solubility data, the thermodynamic solution, mixing, and transfer functions were calculated in addition to performing the enthalpy–entropy compensation analysis. The solubility of isoniazid depends on the concentration of PEG 200 (positive cosolvent effect) and temperature (endothermic process) reaching its maximum solubility in pure PEG 200 at 318.15 K and the lowest solubility in pure water at 278.15 K. The solution process is favored by the solution entropy and according to the enthalpy–entropy compensation analysis it is driven by entropy in mixtures rich in water and by enthalpy in mixtures rich in PEG 200.

## 1. Introduction

**Figure 1:**
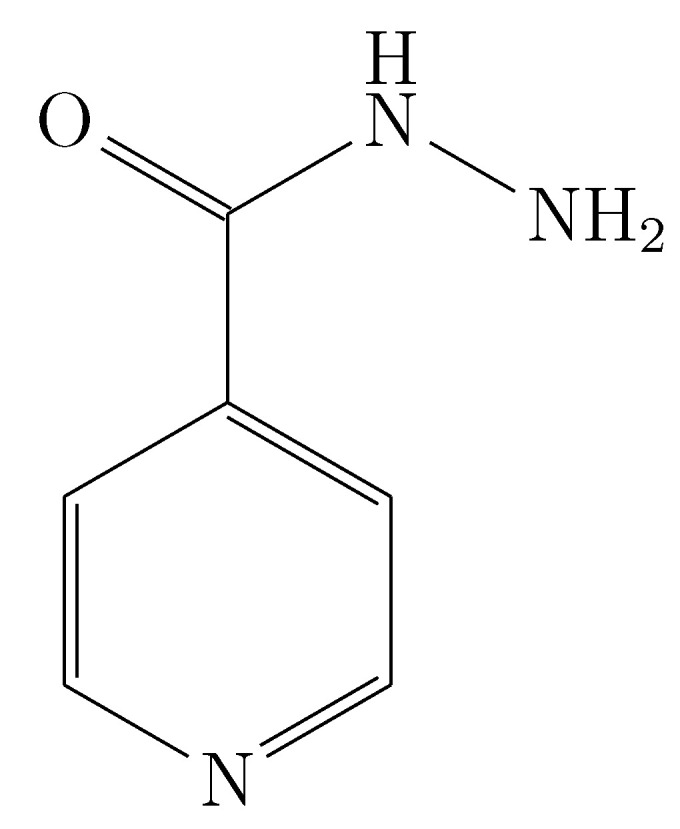
Molecular structure of isoniazid.

Isoniazid (Figure 1, INH, C${}_{6}$H${}_{7}$N${}_{3}$O; IUPAC nomenclature: pyridine-4-carbohydrazide) is an antibiotic primarily used in the treatment of mycobacterial infections. It was first used in 1952 for the treatment of tuberculosis (TB) and remains to this day the choice of treatment for this disease [1]. INH is currently used for the treatment of TB as part of combination therapy or as monotherapy for latent TB, although it may also be used in the treatment of nontuberculous mycobacterial infections [2]. INH is a highly effective bactericidal agent, selective for mycobacteria, because it is a prodrug that requires activation by a mycobacterial-specific catalase. Once activated, INH interferes with cell wall synthesis by inhibiting the synthesis of mycolic acids, an essential component of the bacterial cell wall. Due to its effective role in preventing active TB in at-risk patients [3] and treating active TB [4], INH is a relevant drug that deserves further study, because isoniazid remains a first-line drug for the management of tuberculosis along with other drugs [5].

Solubility is one of the most important parameters in many processes, such as the discovery of new active pharmaceutical ingredients (APIs) [6], the evaluation of bioavailability, the development of formulations [7], and the design of efficient chemical processes (crystallization, purification, synthesis, and quantification) [8].

Cosolvency is one of the most widely used techniques to increase the solubility of poorly soluble APIs, through at least two possible mechanisms: change in the polarity of the cosolvent system and destructuring of the water around the nonpolar groups of the API (hydrophobic hydration) [9,10,11].

The solubility of INH has been studied in some pure solvents such as water (W), methanol, ethanol, n-propanol, isopropyl alcohol, dimethylformamide, ethyl acetate, acetonitrile, and acetone [12,13] and in some cosolvent mixtures of pharmaceutical interest such as (methanol + water), (ethanol + water) and (isopropyl alcohol + water) [12]. Nevertheless, it is clear that the amount of information reported on the solubility of INH is small, so it is necessary to strengthen the database by reporting the solubility of this API in other pharmaceutically relevant solvents such as polyethylene glycol 200 (PEG 200). Since polyethylene glycol is one of the organic solvents with the greatest prospective use, due to properties such as a low volatility, which allows a better volume control, reduces the risk of explosion, and minimizes inhalation effects. On the other hand, since it is miscible with water in all proportions [14,15], PEG has great potential to be used as a cosolvent to increase the solubility of drugs with a low aqueous solubility [16,17], since it has the capacity to dissolve a wide range of substances. An important characteristic of PEGs is that they are nontoxic, environmentally friendly, and biodegradable solvents, which is why they are considered green solvents [18,19]. An equally important fact is that, due to its high industrial production, PEG is a relatively less expensive solvent. These properties make PEG solvents of great use in the pharmaceutical, cosmetic, and food industries [20,21].

Therefore, in this work the solubility of INH in (PEG 200 + water) cosolvent mixtures at nine temperatures is reported, carrying out a thermodynamic and enthalpic–entropic compensation analysis, with the purpose of contributing to the understanding of the possible molecular interactions that occur in the INH solution process.

## 2. Results and Discussion

### 2.1. Experimental Solubility ($x_{3}$)

**Figure 2:**
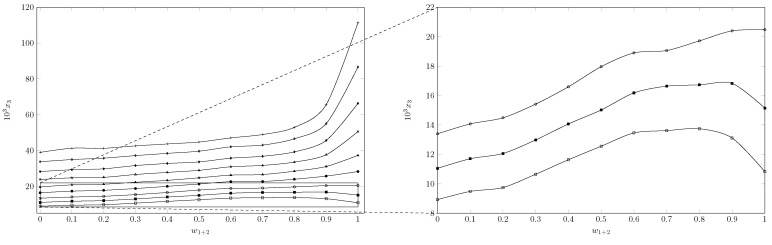
Molar fraction of isoniazid (10${}^{3}$$x_{3}$) depending on the cosolvent composition (mass fraction) free of solute. □: 278.15 K; ■: 283.15 K; ∘: 288.15 K; •: 293.15 K; ▲: 298.15 K; Δ: 303.15 K; ♦: 308.15 K; ⋄: 313.15 K, and +: 318.15 K.

**Figure 3:**
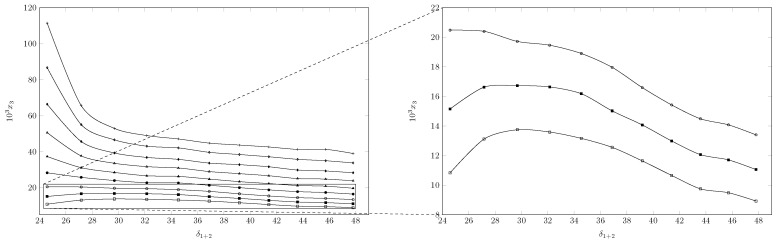
Molar fraction of isoniazid (10${}^{3}$$x_{3}$) depending on the solubility parameter of the cosolvent mixture free of solute. □: 278.15 K; ■: 283.15 K; ∘: 288.15 K; •: 293.15 K; ▲: 298.15 K; Δ: 303.15 K; ♦: 308.15 K; ⋄: 313.15 K, and +: 318.15 K.

Table 1 records the experimental solubility data of INH (substance 3) in (PEG 200 + W) cosolvent mixtures expressed in molar fraction ($x_{3}$). The solubility of INH increases with increasing temperature in all cases, reaching the lowest solubility in pure water at 278.15 K and the highest solubility in pure PEG 200 at 318.15 K. As seen in Figure 2 and Figure 3, the effect of temperature is less in mixtures rich in water and intermediate or greater in mixtures rich in PEG 200.

When analyzing the cosolvent effect, it is observed that at 278.15 K and 283.15 K the maximum solubility is reached in the cosolvent mixture with $w_{1}=0.8$ and $w_{1}=0.9$, respectively; as the temperature increases, the maximum solubility is reached in pure PEG 200 (Figure 2 and Figure 3). In general terms, between 278.15 K and 288.15 K the solubility of isoniazid increases approximately 1.5 times, changing the cosolvent composition where the maximum solubility is reached; from 288.15 K to 318.15 K, the cosolvent effect of PEG 200 is positive in all cases and increases linearly, achieving its greatest effect at 318.15 K where the solubility of isoniazid increases 2.9 times due to the addition of PEG 200.

**Figure 4:**
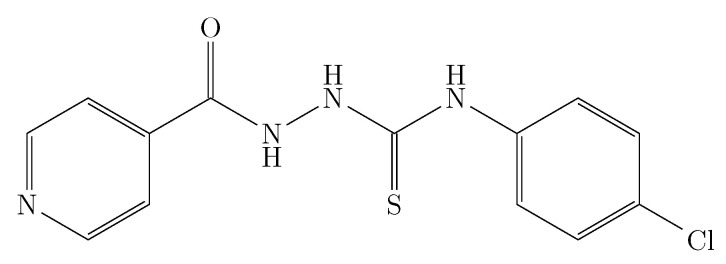
Molecular structure of the INH analog.

Bhat et al. reported the solubility of an INH analog, N-(4-chlorophenyl)-2-(pyridin-4-ylcarbonyl) hydrazinecarbothioamide (Figure 4) [16,17]. When comparing the aqueous solubility of INH and the INH analog, it was observed that the INH analog had a solubility $3.8\times 10^{5}$ times lower than INH; this may be due to solid state properties such as the enthalpy of fusion. Delgado et al. studied the solubility of three structurally related sulfonamides (sulfadiazine (SD), sulfamethazine (SMT), and sulfamerazine (SMT)) in three cosolvent systems (methanol + water; ethanol + water, and 1-propanol + water) [22,23,24,25,26,27]. When comparing the solubility of the three sulfonamides in a solvent, it was possible to establish that the solubility was related to the enthalpy of fusion, thus, SD ($\Delta_{\mathrm{fus}}H=44.3\pm 0.4$ kJ/mol [23]) was less soluble than SMR ($\Delta_{\mathrm{fus}}H=41.3\pm 1.0$ kJ/mol [25]) and it in turn was less soluble than SMT ($\Delta_{\mathrm{fus}}H=39.2\pm 0.7$) kJ/mol [26]). On the other hand, when comparing the solubility of INH in PEG 200 and that of the INH analog in PEG 400, the solubility of INH was three times lower than that of the INH analog; in this case there are two possible causes, the first one was described above (properties of the solid state) and the second one is related to the properties of the solvent. In the study of Delgado et al., it could be established that the solubility was related to the solubility parameter of the compounds; the greater the similarity between the solubility parameter of the solute and the solvent, the more favored the solubility of the drug; therefore, when comparing the solubility of the INH analog in PEG 400 and PG, it can be established that the INH analog, possibly presents a solubility parameter closer to PEG 400 (23.10 MPa${}^{1/2}$ [28]) than to PG (30.7 MPa${}^{1/2}$ [29]), since the solubility of the INH analog is higher in PEG 400.

Figure 3 shows the effect of the polarity of the cosolvent medium on the solubility of the drug; when calculating the solubility parameter of isoniazid using Fedor’s method [29] (Table 2), a solubility parameter of 30.54 MPa${}^{1/2}$ was obtained, which was similar to the solubility parameter of the cosolvent mixture $w_{1}$ = 0.8, whose solubility parameter was 29.7 MPa${}^{1/2}$ and was where the maximum solubility was reached at 278.15 K. However, when increasing the temperature to 283.15 K, the point of maximum solubility moved to $w_{1}$ = 0.9, whose calculated solubility parameter was 27.16 MPa${}^{1/2}$. From 283.15 K, the maximum solubility was reached in PEG 200 whose solubility parameter was 24.58 MPa${}^{1/2}$ [30,31]. This could be due to the influence of temperature on Keesom interactions, which in turn would decrease the polarity of the system [32,33], especially in mixtures rich in PEG 200, which, due to its molecular size and molecular structure compared to water, would be more affected, which in turn would agree with the increase in the positive cosolvent effect of PEG 200 as the temperature increases.

In order to verify possible polymorphic changes that influence the solubility of isoniazid, three differential scanning calorimetry tests were performed on three samples of the drug in equilibrium with the solvent and contrasted with the DSC (differential scanning calorimetry) of the original sample.

**Figure 5:**
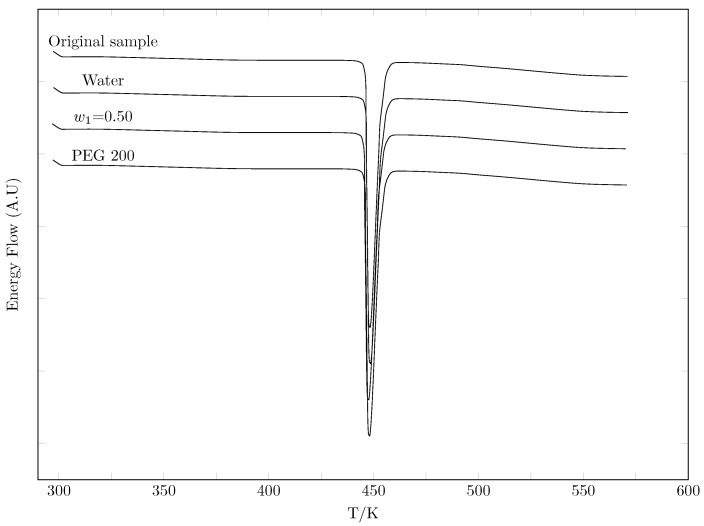
DSC Spectra of Isoniazid.

Figure 5 shows the DSC spectra of the three INH samples in equilibrium with the solvents and the DSC of the original INH sample. It can be verified that the solid phase is the same in all cases, indicating that no polymorphic transition occurs due to the solvent.

Table 3 presents the results of the DSC analysis, that is, the melting, enthalpy data, and the melting temperature data of the four analyzed samples, which agree with the results reported by Forte et al. [34], Duarte et al. [35], and Gong et al. [12].

**Figure 6:**
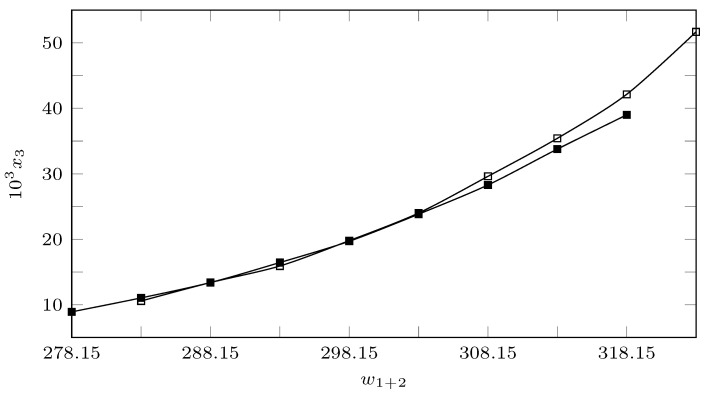
Experimental solubility of isoniazid in pure water □: Gong et al. [12]; ■: this work.

Gong et al. [12] reported the solubility data of INH in pure water at eight temperatures (283.15–323.15 K). When comparing their results with those obtained in this work (Figure 6), a good correlation was observed with most of the data, presenting percentage differences of less than 1.0% at 288.15, 298.15, and 303.15 K; at 283.15, 308.15, and 313.15 K, the percentage difference was less than 5% and at 318.15 K, where the greatest difference between the data occurred, the difference was 8.1%.

### 2.2. Ideal Solubility and Activity Coefficients

The activity coefficient ($\gamma_{3}$) allows the evaluation of the possible molecular interactions that may occur in the dissolution process of INH in the cosolvent system.

For the calculation of $\gamma_{3}$, the ideal solubility must be calculated, which only depends on the physicochemical properties of INH, that is, it only involves the energy required to break the crystalline structure of the solute to melt and subsequently dissolve [36]. Thus, the ideal solubility of INH was calculated according to the Equation (1)

(1) $$\ln x_{3}^{id}=-\frac{\Delta_{m}H}{R}\left(\frac{T_{m}-T}{T_{m}T}\right)+\frac{\Delta C_{p}}{R}\left(\frac{T_{m}-T}{T}\right)-\frac{\Delta Cp}{R}\ln\left(\frac{T_{m}}{T}\right)$$

where *T* and $T_{m}$ are in K, $\Delta_{m}H$ is the enthalpy of fusion (in kJ mol${}^{-1}$) of the solute, *R* is the gas constant (in kJ mol${}^{-1}$K${}^{-1}$), and $\Delta C_{p}$ is the differential heat capacity of fusion (in kJ K${}^{-1}$mol${}^{-1}$) [36]. Some researchers such as Hildebrand and Scott [37], Neau and Flynn [38], Neau et al. [39], and Opperhuizen et al. [40], assume $\Delta C_{p}$ as the entropy of fusion ($\Delta_{m}S$), which is calculated as $\Delta_{m}H/T_{m}$.

Once $x_{3}^{id}$ was calculated at the different study temperatures, $\gamma_{3}$ was calculated from the data of $x_{3}$ (Table 1) as:

(2) $$\gamma_{3}=\frac{x_{3}^{id}}{x_{3}}$$

Using Equation (3), the results obtained with Equation (2), can be analyzed, in terms of molecular solute–solute ($e_{33}$), solute–solvent ($e_{13}$), and solvent–solvent ($e_{11}$) interactions.

(3) $$\ln\gamma_{3}=\left(e_{11}+e_{33}-2e_{13}\right)\frac{V_{3}\phi_{1}^{2}}{RT}$$

In this context, Table 4 shows the activity coefficients of INH in (PEG 200 + W) cosolvent mixtures at different temperatures.

It is observed that as the temperature increases, the activity coefficient decreases, indicating a possible increase in molecular interactions $e_{13}$, which in general terms favors the solution process. Regarding the cosolvent effect on $\gamma_{3}$, except for the results of $x_{3}$ at 278.15 K and 283.15 K where the values of $\gamma_{3}$ closest to ideality are found in a cosolvent mixture, the addition of PEG 200 to the system also favors the $e_{13}$ interactions. In general terms, the solubility of INH in cosolvent mixtures (PEG 200 + W) presents a quasi-ideal behavior, since the values of $\gamma_{3}$ are very close to 1.0.

### 2.3. Thermodynamic Functions of Solution

The thermodynamic solution functions (Table 5) were calculated from experimental solubility data (Table 1), following the Gibbs–van ’t Hoff approach, reformulated by Krug according to the following expressions [41,42]:

(4) $$\Delta_{\mathrm{soln}}H^{o}=-R{\left(\frac{\partial \ln x_{3}}{\partial \left(T^{-1}-T_{\mathrm{hm}}^{-1}\right)}\right)}_{p}$$

(5) $$\Delta_{\mathrm{soln}}G^{o}=-RT_{\mathrm{hm}}.\mathrm{intercept}$$

(6) $$\Delta_{\mathrm{soln}}S^{o}=\left(\Delta_{\mathrm{soln}}G^{o}-\Delta_{\mathrm{soln}}G^{o}\right)T_{\mathrm{hm}}^{-1}$$

(7) $$\zeta_{H}=|\Delta_{\mathrm{soln}}H^{o}|(|T\Delta_{\mathrm{soln}}S^{o}\left|+\right|\Delta_{\mathrm{soln}}S^{o}{\left|\right)}^{-1}$$

(8) $$\zeta_{TS}=1-\zeta_{H}$$

Here, $\Delta_{\mathrm{soln}}H^{o}$, $\Delta_{\mathrm{soln}}G^{o}$, and $\Delta_{\mathrm{soln}}S^{o}$ are the thermodynamic functions (in kJ mol${}^{-1}$) of the enthalpy, Gibbs energy, and entropy of the solution. $T_{\mathrm{hm}}$ is the harmonic temperature (in K), *R* is the gas constant (kJ mol${}^{-1}$K${}^{-1}$), and $\zeta_{H}$ y $\zeta_{TS}$ are the contributions of enthalpy and entropy to the Gibbs energy.

**Figure 7:**
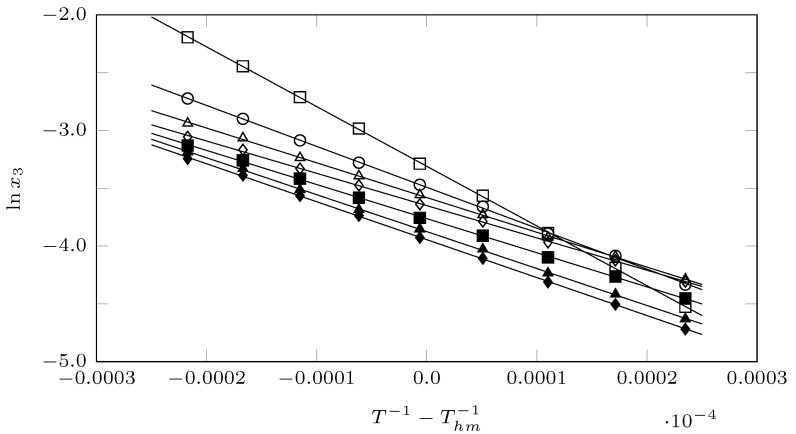
van ’t Hoff plot, for isoniazid (3) in (PEG 200 (1) + water (2)) cosolvent mixtures at some cosolvent mixtures, ♦: $w_{1}=0.0$; ▲: $w_{1}=0.2$; ■: $w_{1}=0.4$; ⋄: $w_{1}=0.6$; △: $w_{1}=0.8$; ∘: $w_{1}=0.9$; □: $w_{1}=1.0$.

From Figure 7, which shows a linear relationship between the $\ln x_{3}$ and $T^{-1}-T_{\mathrm{hm}}^{-1}$ with $r^{2}\approx =0.990$, $\Delta_{\mathrm{soln}}H^{o}$ and $\Delta_{\mathrm{soln}}G^{o}$ can be determined according to Equations (4) and (5).

In all cases, $\Delta_{\mathrm{soln}}G^{o}$ is positive and decreases with the increase in the concentration of PEG 200 in the cosolvent mixture, as a consequence of the increase in the solubility of INH; $\Delta_{\mathrm{soln}}H^{o}$ is also positive, which indicates that the solution process of INH in the (PEG 200 + W) cosolvent system is endothermic. The enthalpy values of solution decrease from pure water to the cosolvent mixture $w_{1}$ = 0.6, which implies a flourishing of solute–solvent molecular interactions ($e_{13}$), increasing the solubility of INH. From $w_{1}$ = 0.6 to $w_{1}$ = 1.0, an increase in enthalpy occurs, possibly due to the formation of self-aggregations of PEG 200 in PEG 200-rich mixtures [33]; as for $\Delta_{\mathrm{soln}}S^{o}$, it is positive, which favors the solution process. Like enthalpy, solution entropy decreases from pure water to $w_{1}$ = 0.6 and increases from this PEG 200 concentration to pure PEG 200.

From the results of Equations (7) and (8), it is concluded that the energy component ($\zeta_{H}$) predominates over the organizational component ($\zeta_{TS}$), contributing in all cases in more than 55%.

**Figure 8:**
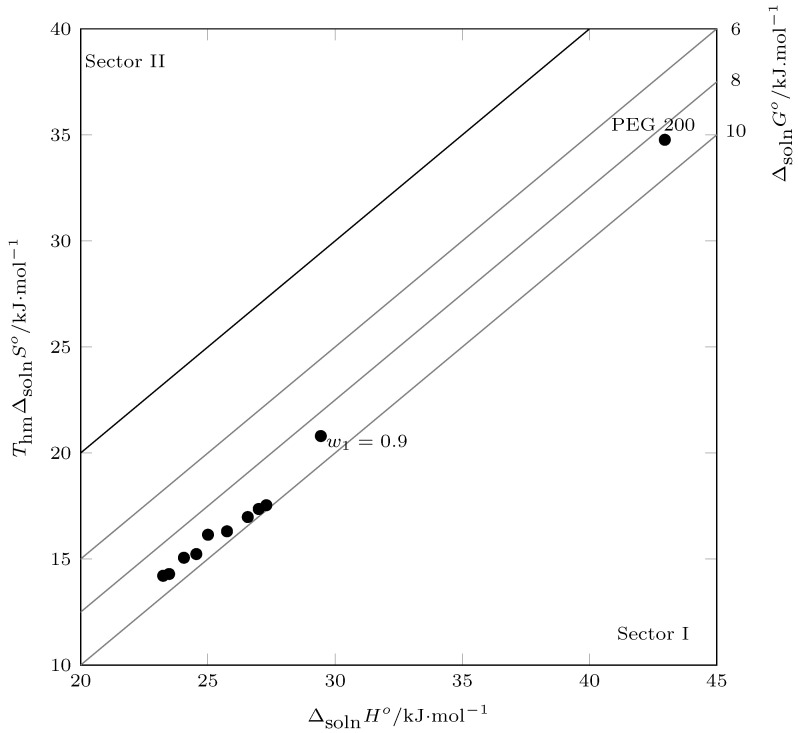
Relation between enthalpy ($\Delta_{\mathrm{soln}}H^{o}$) and entropy ($T_{\mathrm{hm}}\Delta_{\mathrm{soln}}S^{o}$) in terms of the process of isoniazid (3) solution in (PEG 200 (1) + water (2)) cosolvent mixtures at 297.6 K. The isoenergetic curves for $\Delta_{\mathrm{soln}}G^{o}$ are represented by dotted lines.

According to the Perlovich graph (Figure 8), all data were recorded in sector I ($\Delta_{\mathrm{soln}}H^{0}>T\Delta_{\mathrm{soln}}S^{o}$), indicating that the solution process was enthalpy-driven, as well as entropy-favored [43,44].

### 2.4. Thermodynamic Functions of Transfer

In Table 6, the thermodynamic transfer functions calculated according to Equation (9) are reported. Where *f* represents the Gibbs energy, enthalpy or transfer entropy.

In this case, the transfer process is a hypothetical process between miscible solvents, which allows the evaluation of the influence of the cosolvent PEG 200 in the dissolution process of INH. Each thermodynamic function was calculated by subtracting the thermodynamic quantity of the medium of higher polarity from that of lower polarity.

(9) $$\Delta_{\mathrm{tr}}f^{o}=\Delta_{\mathrm{soln}}f_{\mathrm{less}\;\mathrm{polar}}^{o}-\Delta_{\mathrm{soln}}F_{\mathrm{more}\;\mathrm{polar}}^{o}$$

Considering the addition of PEG 200 to water, which in principle decreases the polarity of the cosolvent system, the following occurred: from $w_{1}=0.0$ (δ=47.8 MPa${}^{1/2}$ [29]) to $w_{1}=0.6$ (δ=34.54 MPa${}^{1/2}$), $\Delta_{\mathrm{tr}}G^{0}$ was negative, indicating that the hypothetical transfer process from pure water to any cosolvent mixture ≤$\left(w_{1}=0.6\right)$ occurred, further indicating that PEG 200 favored the solution process, which was favored by $\Delta_{\mathrm{tr}}H^{0}$ (negative) and disadvantaged by $\Delta_{\mathrm{tr}}S^{0}$ (negative); from $w_{1}=0.6$ to $w_{1}=1.0$ (δ=24.58 MPa${}^{1/2}$), $\Delta_{\mathrm{tr}}G^{0}$ was negative, so the transfer process was also energetically viable; however, in this composition range the process was favored by $\Delta_{\mathrm{tr}}S^{0}$ (positive) and disadvantaged by $\Delta_{\mathrm{tr}}H^{0}$ (positive).

**Figure 9:**
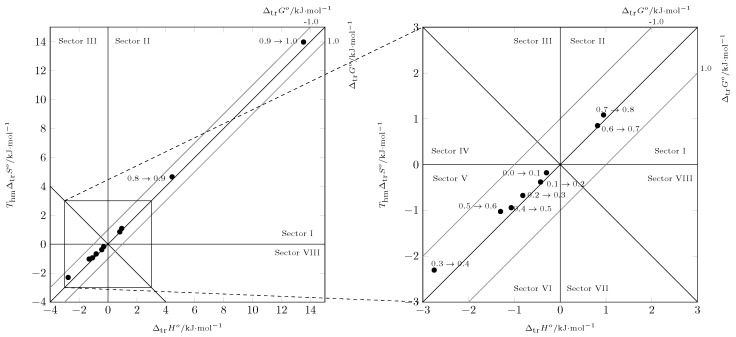
Relation between enthalpy ($\Delta_{\mathrm{tr}}H^{o}$) and entropy ($T_{\mathrm{hm}}\Delta_{\mathrm{tr}}S^{o}$) of the process transfer of isoniazid (3) in (PEG 200 (1) + water (2)) cosolvent mixtures at 297.6 K. The isoenergetic curves for $\Delta_{\mathrm{mix}}G^{o}$ are represented by dotted lines.

Analyzing the process using Perlovich’s graphic method [43,44] (Figure 9) from $w_{1}=0.0$ (δ=47.8 MPa${}^{1/2}$ [29]) to $w_{1}=0.6$ (δ=34.54 MPa${}^{1/2}$), the data were recorded in sector V ($\Delta_{\mathrm{soln}}H^{0}<0$ and $T\Delta_{\mathrm{soln}}S^{0}<0$) and from $w_{1}=0.6$ (δ=34.54 MPa${}^{1/2}$) to pure PEG 200, the data were recorded in sector I ($\Delta_{\mathrm{soln}}H^{0}>T\Delta_{\mathrm{soln}}S^{0}$), indicating that in all cases the transfer process was driven by the transfer enthalpy.

### 2.5. Thermodynamic Functions of Mixing

The solution process involves the change of state of the solute (Solute${}_{\mathrm{solid},T}$→ Solute ${}_{\mathrm{solid},T_{m}}$ → Solute ${}_{\mathrm{liquid},T_{m}}$ → Solute ${}_{\mathrm{liquid},T}$), the molecular rearrangement of solvent to form a cavity that allows the solute to lodge, and finally the process of mixture, which consists of the molecular interaction between the solute and the solvent to form the solution (Solute${}_{\mathrm{liquid},T}$ → Solute ${}_{\mathrm{soln}}$) [45].

The solution process can be described by Equation (10)

(10) $$\Delta_{\mathrm{Sol}}f^{o}=\Delta_{\mathrm{mix}}f^{o}+\Delta_{m}f^{o}$$

Clearing $\Delta_{\mathrm{mix}}f^{o}$ in (10), we get:

(11) $$\Delta_{\mathrm{mix}}f^{o}=\Delta_{\mathrm{soln}}f^{o}-\Delta_{m}f^{o}$$

Therefore, using Equation (11), the thermodynamic mixing functions were calculated.

Table 7 shows the thermodynamic mixing functions of INH. The Gibbs energy of mixing is positive in all cases and decreases from pure water to pure PEG 200, although the mixing process is unfavorable to the solution process, and the addition of PEG 200 to the cosolvent system promotes solute–solvent interactions. As for the enthalpy of mixture, it is also positive in all cases, and it decreases from pure water to $w_{1}=0.6$ and increases from this cosolvent mixture to PEG 200, where it reaches its highest value. The decrease in the enthalpy of the mixture in intermediate mixtures may be due to the fact that the PEG 200 – W interactions are less energetic than the W–W, and PEG 200–PEG 200 interactions; therefore, the formation of the cavity to house the solute is energetically more viable in intermediate mixtures. Finally, the mixing entropy is positive in all cases, indicating a favoring of the mixing process and, therefore, of the solution process.

**Figure 10:**
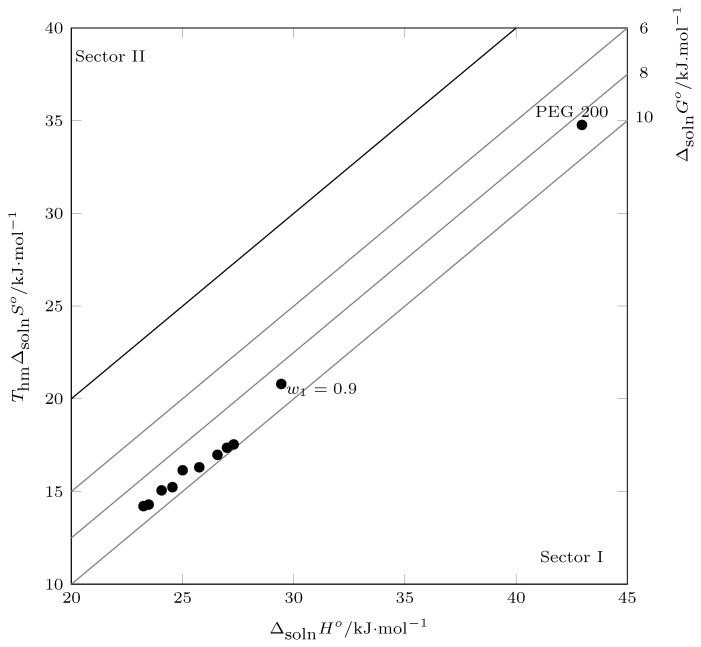
Relation between enthalpy ($\Delta_{\mathrm{mix}}H^{o}$) and entropy ($T_{\mathrm{hm}}\Delta_{\mathrm{mix}}S^{o}$) of the process mixing of isoniazid (3) in (PEG 200 (1) + water (2)) cosolvent mixtures at 297.6 K. The isoenergetic curves for $\Delta_{\mathrm{mix}}G^{o}$ are represented by dotted lines.

When performing Perlovich’s graphical analysis [43,44] (Figure 10), the values of the thermodynamic mixing functions were recorded in sector I ($\Delta_{\mathrm{soln}}H^{0}>T\Delta_{\mathrm{soln}}S^{0}$), indicating that the mixing process was driven by the enthalpy of mixing.

### 2.6. Enthalpy–Entropy Compensation Analysis

The enthalpy–entropy compensation analysis can be performed by plotting $\Delta_{soln}H^{o}$ vs. $\Delta_{soln}G^{o}$, where positive slopes indicate enthalpy conduction and negative slopes indicate entropic conduction [46,47], or plotting $\Delta_{soln}H^{o}$ vs. $T\Delta_{soln}S^{o}$, where slopes greater than 1.0 indicate enthalpy conduction and slopes less than 1.0 indicate entropic conduction [48].

**Figure 11:**
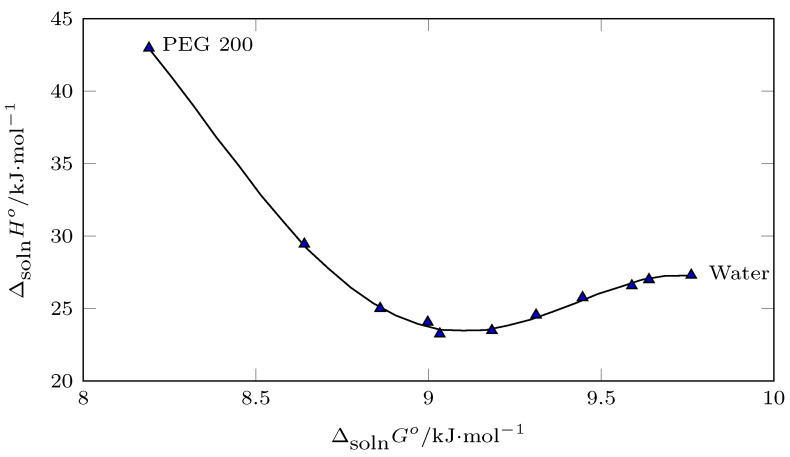
Enthalpy–entropy compensation plot for the solubility of isoniazid (3) in (PEG 200 (1) + water (2)) mixtures at $T_{hm}$ = 297.6 K.

**Figure 12:**
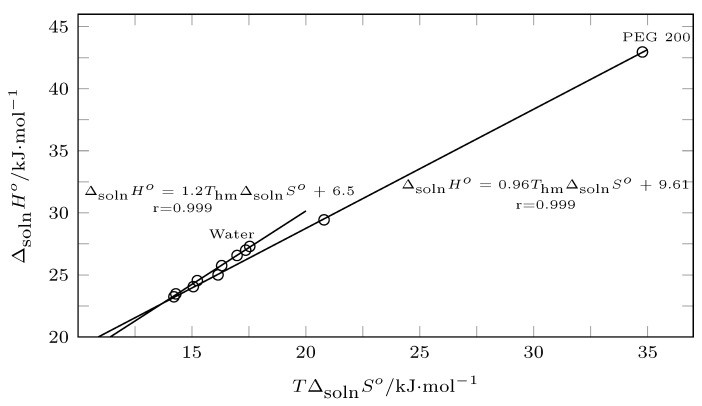
Enthalpy–entropy compensation plot for the solubility of isoniazid (3) in (PEG 200 (1) + water (2)) mixtures at $T_{hm}$ = 297.6 K.

In this context, from pure water to $w_{1}=0.60$, the process was driven by enthalpy according to Figure 11 (positive slope), which was corroborated by Figure 12 (slope > 1.0), and from $w_{1}=0.60$ to PEG 200, the solution process was driven by entropy (Figure 11, negative slope; Figure 12 slope < 1.0).

Thus, in mixtures rich in water, the addition of PEG 200 promoted the formation of solute–solvent bonds, so the energy component ($\Delta_{soln}H^{o}$) promoted an increase in the solubility of INH in terms of mixtures rich in PEG 200. Although there was an increase in $\Delta_{soln}H^{o}$, the organizational factor ($T\Delta_{soln}S^{o}$) was the one that governed the solution process, that is, by increasing the concentration of PEG 200, both the water and the PEG 200 tended to break down and interact with INH.

### 2.7. Preferential Solvation

A preferential solvation analysis allows the assessment of the possible interactions that occur at the molecular level between the solute molecules and the solvents that make up the cosolvent mixture. This analysis can be done by using the inverse Kirkwood–Buff integral (IKBI) method proposed by Ben-Naim, from which the preferential solvation parameters $\delta x_{1,3}$ and $\delta x_{2,3}$ are calculated, which indicate the tendency of the solute (3) to be solvated by solvents 1 or 2 [49].

Ben-Naim developed the IKBI model, presenting the following equations [50,51]:

(12) $$\delta x_{1,3}^{o}=x_{1,3}^{L}-x_{1}=-\delta x_{2,3}^{o}$$

(13) $$\delta x_{1,3}^{o}=\frac{x_{1}x_{2}\left(G_{1,3}-G_{2,3}\right)}{x_{1}G_{1,3}+x_{2}G_{2,3}+V_{\mathrm{cor}}}$$

(14) $$G_{1,3}=RT\kappa_{T}-V_{3}+x_{2}V_{2}DQ^{-1}$$

(15) $$G_{2,3}=RT\kappa_{T}-V_{3}+x_{1}V_{1}DQ^{-1}$$

(16) $$V_{\mathrm{cor}}=2522.5{\left\{r_{3}+0.1363{\left(x_{1,3}^{L}V_{1}+x_{2,3}^{L}V_{2}\right)}^{1/3}-0.085\right\}}^{3}$$

(17) $$D={\left(\frac{\partial \Delta_{\mathrm{tr}}G_{3,1-->1+2}^{0}}{x_{1}}\right)}_{T,P}$$

(18) $$Q=RT+x_{1}x_{2}{\left(\frac{\partial^{2}G_{1,2}^{Exc}}{x_{2}^{2}}\right)}_{T,P}$$

$G_{1,3}$ and $G_{2,3}$ are the Kirkwood–Buff integrals (in cm${}^{3}$/mol), $V_{\mathrm{cor}}$ is the correlation volume around solute (3) within which preferential solvation takes place (in cm${}^{3}$/mol), $\kappa_{T}$ is the isothermal compressibility of the mixtures (in ${\mathrm{GPa}}^{-1}$), $V_{3}$, $V_{1}$, and $V_{2}$ are the partial molar volumes of the solute and solvents (in cm${}^{3}$/mol), and *D*, *Q*, and RT (in kJ/mol) are expressed in Equations (17) and (18). The $\kappa_{T}$ of the cosolvent mixture was calculated as: $x_{1}\kappa_{T,1}+x_{2}\kappa_{T,2}$ and the partial molar volumes were calculated from the density data reported by Yasmin et al. [52]. The correlation volume was calculated by iteration due to dependency on the local mole fractions ($x_{1,3}^{L}$ and $x_{2,3}^{L}$) given by Equations (12) and (16).

Table 8 shows the values of the different variables necessary for the calculation of the preferential solvation parameters. The transfer Gibbs energy of INH from the water to each PEG 200 + W cosolvent mixture was calculated from the INH solubility data according to:

(19) $$\Delta_{tr}G_{3,2->1+2}^{0}=RT\ln\left(\frac{x_{3,2}}{x_{3,1+2}}\right)=\left(\frac{-0.004-11.965x_{1}+-1.449x_{1}^{2}}{1+10.288x_{1}-2.830x_{1}^{2}}\right)$$

**Figure 13:**
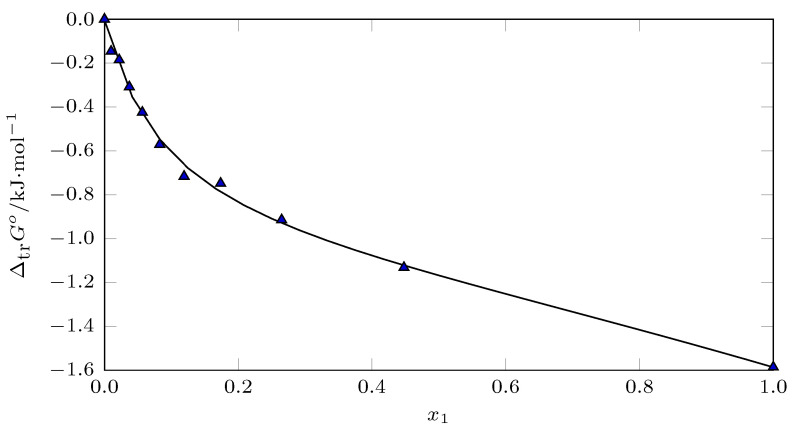
Gibbs energy of transfer of isoniazid (3) from neat water to (PEG 200 (1) + water (2)) cosolvent mixtures at *T* = 298.15 K.

From the $\Delta_{tr}G_{3,2->1+2}^{0}$ data (Figure 13), the *D* values were calculated. On the other hand, the *Q* values were calculated from the $G_{1,2}^{Exc}$ data reported by Ninni et al. [53].

For the calculation of the Kirkwood–Buff integrals, the values of $\kappa_{T}$ PEG 200 0.42 ${\mathrm{GPa}}^{-1}$ [52] and water 0.457 ${\mathrm{GPa}}^{-1}$ [54] were taken, $V_{3}$ was taken as 108.69 cm${}^{3}$/mol [55], and $V_{1}$ and $V_{2}$ were calculated from the density data reported by Muñoz et al. [56] according to Equations (20) and (22).

(20) $$\bar{V_{1}}=V+x_{2}\frac{dV}{dx_{1}}$$

(21) $$\bar{V_{2}}=V+x_{1}\frac{dV}{dx_{2}}$$

The value of $r_{3}$ (0.351 nm), necessary for the calculation of $V_{cor}$, was calculated from $V_{3}$ (Equation (22)).

(22) $$r_{3}=\left(\frac{3\times 10^{21}V_{3}}{4\pi N_{Av}}\right)$$

Here, $N_{Av}$ is the Avogadro number.

According to the results of the Kirkwood–Buff integrals, where negative values were obtained in all cases, it can be inferred that INH had affinity for the two solvents (PEG and W) [57].

**Figure 14:**
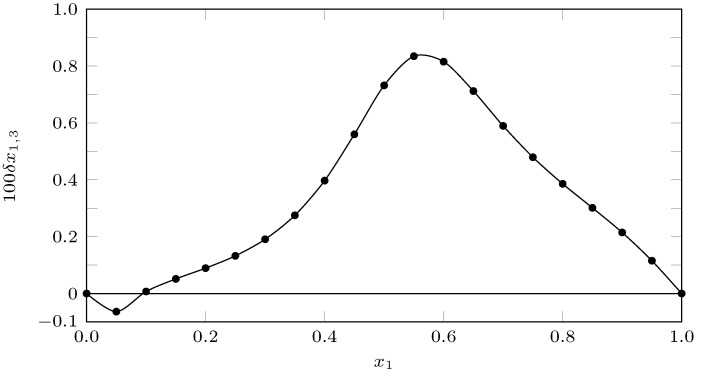
$\delta x_{1,3}$ values for the isoniazid (3) (PEG 200 (1) + water (2)) cosolvent mixtures at 298.15 K.

In relation to the values of $\delta x_{1,3}$ (Table 8, Figure 14), from pure water up to $x_{1}=0.1$, the INH tended to be solvated by water; from $x_{1}=0.1$ up to pure PEG 200, the INH tended to be solvated by PEG 200; however, in all cases, the values of $\delta x_{1,3}$ were less than 0.01, so the results could be a consequence of the propagation of uncertainties in the calculations of the inverse integrals of Kirkwood–Buff rather than a real process of solvation by one of the solvents [49,58].

This may have occurred because the increase in solubility as a consequence of increasing the concentration of PEG 200 in the cosolvent mixture was relatively low, which could also be verified by reviewing the values of the thermodynamic functions of transfer, which showed that the addition of PEG 200 did not lead to a significant increase in the solubility of INH.

## 3. Materials and Methods

### 3.1. Reagents

In this study, isoniazid (Sigma-Aldrich, Burlington, MA, USA; compound **3**, with purities of at least 0.990 in mass fraction), polyethylene glycol 200 (Sigma-Aldrich, Burlington, MA, USA; the solvent component 1, purity of at least 0.998 in mass fraction) were used. Table 9 summarizes the sources and purities of the compounds studied.

### 3.2. Preparation of Solvent Mixtures

Nine cosolvent mixtures of PEG 200 (substance 1) and water (substance 2) were prepared gravimetrically (mass fraction) from 0.1 to 0.9 using an analytical balance with sensitivity of ±0.0001 g (RADWAG AS 220.R2, Toruń, Poland). Each of the cosolvent systems was prepared in an amber glass bottle with a capacity of 15 mL; 3 samples of 10.00 ± 0.00 g were prepared independently for each gravimetric concentration.

### 3.3. Solubility Determination

Isoniazid solubility was determined according to the shake-flask method proposed by Higuchi and Connors [59].

Sufficient isoniazid was added to each of the cosolvent samples to saturate them and obtain an excess of undissolved drug. Each sample was subjected to ultrasound for 10 min in order to reduce the particle size of the isoniazid sample and increase the dissolution rate. Once the samples were saturated (it was verified that they all presented two phases: saturated solution (liquid phase) and undissolved drug (solid phase) they were placed in a recirculating water bath (Medingen K-22/T100, Dresden, Germany) at each one of the study temperatures (278.15, 283.15, 288.15, 293.15, 298.15, 303.15, 308.15, 313.15, and 318.15 K) for 72 h.

After saturating the samples, an aliquot was taken from each of the bottles using a previously thermostated syringe; to avoid the presence of undissolved solid particles, the aliquot was filtered with a membrane with a pore diameter of 0.45 µm (Millipore Corp. Swinnex-13, St. Louis, MO, USA). To avoid possible errors due to drug sorption by the membrane, the filters were purged with saturated solution to saturate possible adsorption sites. Each aliquot was diluted in double-distilled water, and its concentration was determined by UV–vis spectrophotometry (UV/VIS EMC-11-UV spectrophotometer, Dresden, Germany) at 261 nm (wavelength of maximum absorbance).

Each of the experimental solubility data is an average of three repetitions.

### 3.4. Calorimetric Study

The enthalpy and melting temperature of 4 isoniazid samples were determined by differential scanning calorimetry (DSC 204 F1 Phoenix, Dresden, Germany). A mass of approximately 10.0 mg of each sample was deposited in an aluminum crucible and placed in the calorimeter under a nitrogen flow of 10 mL min${}^{-1}$. The heating cycle was developed from 300 to 575 K, with a heating ramp of 10 K min${}^{-1}$.

## 4. Conclusions

The solubility of NHI in PEG 200 + W cosolvent mixtures was an endothermic process and depends on the cosolvent composition. According to the preferential solvation analysis, the NHI molecule showed affinity for both solvents (PEG 200 and W) and its solvation sphere was not mainly composed by a particular solvent.

The solution process was entropy-favored and enthalpy-driven. The mixing process was also favored by entropy and as for the transfer process, it was evident that INH tended to be more soluble in media of less polarity. Finally, according to the enthalpy–entropy compensation analysis, the process was driven by entropy in more polar cosolvent mixtures and by enthalpy in cosolvent mixtures of lower polarity.

## Figures and Tables

**Table 1:** Experimental solubility of isoniazid (3) in (PEG 200 (1) + water (2)) cosolvent mixtures expressed in mole fraction ($10^{3}x_{3}$) at different temperatures. Experimental pressure *p*: 0.1 MPa.

$w_{1}$	Temperature (K)								
	278.15	283.15	288.15	293.15	298.15	303.15	308.15	313.15	318.15
0.0	8.93 ± 0.13	11.05 ± 0.18	13.4 ± 0.21	16.45 ± 0.1	19.7 ± 0.27	23.8 ± 0.5	28.3 ± 0.6	33.8 ± 0.4	39.0 ± 0.4
0.1	9.49 ± 0.07	11.71 ± 0.16	14.08 ± 0.2	17.34 ± 0.34	20.9 ± 0.4	24.8 ± 0.4	29.3 ± 0.23	35 ± 0.6	41.3 ± 0.5
0.2	9.75 ± 0.2	12.06 ± 0.05	14.49 ± 0.14	17.82 ± 0.42	21.23 ± 0.14	25.1 ± 0.4	29.8 ± 0.7	35.8 ± 0.6	41.3 ± 0.6
0.3	10.66 ± 0.08	12.99 ± 0.06	15.42 ± 0.21	18.8 ± 0.12	22.31 ± 0.2	26.65 ± 0.11	31.64 ± 0.29	37.19 ± 0.3	42.6 ± 0.4
0.4	11.64 ± 0.06	14.07 ± 0.07	16.6 ± 0.18	20.04 ± 0.07	23.38 ± 0.25	27.81 ± 0.06	32.81 ± 0.33	38.4 ± 0.4	43.7 ± 0.26
0.5	12.56 ± 0.17	15.02 ± 0.19	17.97 ± 0.27	21.35 ± 0.16	24.8 ± 0.29	28.97 ± 0.3	33.68 ± 0.31	39.7 ± 0.4	44.8 ± 0.7
0.6	13.46 ± 0.30	16.18 ± 0.33	18.9 ± 0.14	22.65 ± 0.23	26.3 ± 0.42	31.0 ± 0.6	35.8 ± 0.4	42.1 ± 0.6	47.1 ± 0.6
0.7	13.39 ± 0.10	16.04 ± 0.14	19.06 ± 0.26	22.75 ± 0.15	26.64 ± 0.34	31.71 ± 0.21	36.83 ± 0.14	43.07 ± 0.3	49.0 ± 0.5
0.8	13.75 ± 0.33	16.73 ± 0.22	19.72 ± 0.3	24.00 ± 0.21	28.49 ± 0.15	33.6 ± 0.6	39.3 ± 1.0	46.6 ± 0.7	53.0 ± 1.0
0.9	13.12 ± 0.18	16.83 ± 0.30	20.4 ± 0.21	25.76 ± 0.33	31.1 ± 0.3	37.7 ± 0.7	45.7 ± 0.5	55.1 ± 1.0	65.6 ± 0.2
1.0	10.83 ± 0.14	15.15 ± 0.41	20.48 ± 0.21	28.3 ± 0.34	37.35 ± 0.57	50.6 ± 0.4	66.4 ± 1.7	86.7 ± 0.6	111.5 ± 0.9

**Table 2:** Isoniazid solubility parameter calculated according to the Fedor’s method.

Group	Group Number	*U* (kJ mol${}^{-1}$)	*V* (cm${}^{3}$ mol${}^{-1}$)
=CH-	4	4.31 × 4 = 17.24	13.5 × 4 = 54
>C=	1	4.31 × 1 = 4.31	−5.5 × 1 = −5.5
-N=	1	11.7 × 1 = 11.7	5.0 × 1 = 5.0
-NH${}_{2}$	1	12.6 × 1 = 12.6	19.2 × 1 = 19.2
-CONH-	1	33.5 × 1 = 33.5	9.5 × 1 = 9.5
Ring closure	1	1.05 × 1 = 1.05	16.0 × 1 = 16.0
Conjugation in ring	3	1.67 × 3 = 5.01	−2.2 × 3 = −6.6
		$U_{T}$ = 85.41	$V_{T}$ = 91.6
		$\delta ={\left(85410/91.6\right)}^{1/2}$ = 30.54 MPa${}^{1/2}$	

**Table 3:** The thermophysical properties of isoniazid obtained by DSC.

Sample	Enthalpy of Fusion, $\Delta_{\mathrm{fus}}H$ (kJ mol${}^{-1}$)	Melting Point $T_{\mathrm{fus}}$ (K)	Ref.
Original sample	28.1 ± 0.5	445.1 ± 0.5	This work
	27.912 ± 0.28	445.84 ± 0.50	[34]
	28.38 ± 0.56	445.15 ± 1.0	[35]
	28.13 ± 1.41	446.04 ± 0.50	[12]
Water	28.4 ± 0.5	446.6 ± 0.5	This work
$w_{0.50}$	27.8 ± 0.5	445.3 ± 0.5	This work
PEG 200	28.1 ± 0.5	446.4 ± 0.5	This work

**Table 4:** Activity coefficient of isoniazid (3) in (PEG 200 (1) + water (2)) cosolvent mixtures at different temperatures and a pressure *p* = 0.096 MPa.

$w_{1}$	Temperature (K)								
	278.15	283.15	288.15	293.15	298.15	303.15	308.15	313.15	318.15
0.0	3.12	2.88	2.71	2.52	2.39	2.24	2.14	2.02	1.98
0.1	2.93	2.72	2.58	2.39	2.26	2.15	2.06	1.95	1.87
0.2	2.85	2.64	2.51	2.32	2.22	2.13	2.03	1.91	1.87
0.3	2.61	2.45	2.36	2.2	2.11	2.01	1.91	1.84	1.81
0.4	2.39	2.26	2.19	2.07	2.01	1.92	1.84	1.78	1.76
0.5	2.22	2.12	2.02	1.94	1.90	1.84	1.80	1.72	1.72
0.6	2.07	1.97	1.92	1.83	1.79	1.73	1.69	1.62	1.64
0.7	2.08	1.98	1.91	1.82	1.77	1.69	1.64	1.59	1.57
0.8	2.02	1.90	1.84	1.73	1.65	1.59	1.54	1.47	1.46
0.9	2.12	1.89	1.71	1.61	1.51	1.42	1.32	1.24	1.17
1.0	2.57	2.10	1.71	1.46	1.26	1.06	0.91	0.79	0.69

**Table 5:** Thermodynamic functions of the solution process of isoniazid (3) in (PEG 200 (1) + water (2)) cosolvent mixtures.

$w_{1}$	$\Delta_{\mathrm{soln}}G^{0}$ (kJ/mol)	$\Delta_{\mathrm{soln}}H^{0}$ (kJ/mol)	$\Delta_{\mathrm{soln}}S^{0}$ (J/mol·K)	$T\Delta_{\mathrm{soln}}S^{0}$ (kJ/mol)	$\zeta_{H}$	$\zeta_{\mathrm{TS}}$
0.0	9.76 ± 0.14	27.3 ± 0.16	58.9 ± 0.9	17.54 ± 0.27	0.609	0.391
0.1	9.64 ± 0.14	27.00 ± 0.15	58.3 ± 0.9	17.36 ± 0.27	0.609	0.391
0.2	9.59 ± 0.14	26.57 ± 0.17	57.1 ± 0.9	16.98 ± 0.28	0.610	0.390
0.3	9.45 ± 0.08	25.75 ± 0.11	54.8 ± 0.5	16.31 ± 0.15	0.612	0.388
0.4	9.31 ± 0.07	24.55 ± 0.11	51.2 ± 0.4	15.24 ± 0.13	0.617	0.383
0.5	9.18 ± 0.11	23.48 ± 0.14	48.0 ± 0.6	14.29 ± 0.19	0.622	0.378
0.6	9.03 ± 0.14	23.24 ± 0.18	47.8 ± 0.8	14.21 ± 0.24	0.621	0.379
0.7	9.00 ± 0.08	24.06 ± 0.11	50.6 ± 0.5	15.06 ± 0.15	0.615	0.385
0.8	8.86 ± 0.14	25.01 ± 0.18	54.3 ± 0.9	16.15 ± 0.28	0.608	0.392
0.9	8.64 ± 0.11	29.44 ± 0.16	69.9 ± 1.0	20.8 ± 0.29	0.586	0.414
1.0	8.19 ± 0.11	42.96 ± 0.16	116.8 ± 1.7	34.77 ± 0.5	0.553	0.447
Ideal	7.58 ± 0.03	18.7 ± 0.17	37.5 ± 0.4	11.17 ± 0.11	0.627	0.373

**Table 6:** Thermodynamic functions of transfer of isoniazid (3) in (PEG 200 (1) + water (2)) cosolvent mixtures at 297.6 K and a pressure *p* = 0.096 MPa.

More Polar→Less Polar	$\Delta_{\mathrm{tr}}G^{0}$ (kJ/mol)	$\Delta_{\mathrm{tr}}H^{0}$ (kJ/mol)	$\Delta_{\mathrm{tr}}S^{0}$ (J/mol·K)	$T\Delta_{\mathrm{tr}}S^{0}$ (kJ/mol)
0.0→0.1	−0.12 ± 0.19	−0.30 ± 0.22	−0.6 ± 1.3	−0.2 ± 0.4
0.1→0.2	−0.05 ± 0.20	−0.40 ± 0.23	−1.3 ± 1.3	−0.4 ± 0.4
0.2→0.3	−0.14 ± 0.16	−0.80 ± 0.21	−2.26 ± 1.1	−0.67 ± 0.31
0.3→0.4	−0.45 ± 0.15	−2.80 ± 0.19	−7.74 ± 1	−2.3 ± 0.3
0.4→0.5	−0.13 ± 0.13	−1.10 ± 0.18	−3.16 ± 0.8	−0.94 ± 0.23
0.5→0.6	−0.28 ± 0.15	−1.30 ± 0.21	−3.4 ± 0.9	−1.02 ± 0.27
0.6→0.7	−0.03 ± 0.16	0.80 ± 0.20	2.86 ± 0.9	0.85 ± 0.28
0.7→0.8	−0.14 ± 0.16	0.90 ± 0.21	3.6 ± 1.1	1.08 ± 0.31
0.8→0.9	−0.22 ± 0.18	4.40 ± 0.24	15.6 ± 1.4	4.7 ± 0.4
0.9→0.10	−0.45 ± 0.16	13.50 ± 0.23	46.9 ± 2.0	14 ± 0.6

**Table 7:** Thermodynamic functions of mixing isoniazid (3) in (PEG 200 (1) + water (2)) cosolvent mixtures at 297.6 K and a pressure *p* = 0.096 MPa.

$w_{1}$	$\Delta_{\mathrm{mix}}G^{0}$ (kJ/mol)	$\Delta_{\mathrm{mix}}H^{0}$ (kJ/mol)	$\Delta_{\mathrm{mix}}S^{0}$ (J/mol·K)	$T\Delta_{\mathrm{mix}}S^{0}$ (kJ/mol)
0.0	2.18 ± 0.14	8.55 ± 0.23	21.4 ± 1.3	6.37 ± 0.29
0.1	2.06 ± 0.14	8.25 ± 0.23	20.8 ± 1.2	6.19 ± 0.29
0.2	2.01 ± 0.15	7.82 ± 0.24	19.5 ± 1.8	5.81 ± 0.3
0.3	1.87 ± 0.08	7.01 ± 0.21	17.3 ± 0.6	5.14 ± 0.19
0.4	1.73 ± 0.07	5.80 ± 0.21	13.7 ± 0.6	4.07 ± 0.17
0.5	1.60 ± 0.11	4.73 ± 0.22	10.5 ± 0.7	3.13 ± 0.22
0.6	1.45 ± 0.14	4.50 ± 0.25	10.2 ± 0.9	3.04 ± 0.27
0.7	1.42 ± 0.08	5.31 ± 0.20	13.1 ± 0.6	3.89 ± 0.18
0.8	1.28 ± 0.14	6.26 ± 0.25	16.7 ± 1.0	4.98 ± 0.3
0.9	1.06 ± 0.12	10.69 ± 0.24	32.4 ± 1.1	9.63 ± 0.31
1.0	0.61 ± 0.12	24.21 ± 0.24	79.3 ± 1.7	23.6 ± 0.5

**Table 8:** Some properties associated to preferential solvation of isoniazid (3) in (PEG 200 (1) + water (2)) cosolvent mixtures.

$x_{1}$	*D*	*Q*	$\mathrm{RT}\kappa_{T}$	$V_{\mathrm{PEG}\;200}$	$V_{W}$	$G_{1,3}$	$G_{2,3}$	$V_{\mathrm{cor}}$	$100\cdot \delta x_{1,3}$
	(kJ/mol)	(kJ/mol)	(cm${}^{3}$/mol)	(cm${}^{3}$/mol)	(cm${}^{3}$/mol)	(cm${}^{3}$/mol)	(cm${}^{3}$/mol)	(cm${}^{3}$/mol)	
0.00	−11.92	2.479	1.133	170.66	18.06	−194.4	−107.5	610	0.00
0.05	−5.37	5.372	1.128	172.40	18.02	−124.6	−116.2	750	−0.06
0.01	−3.17	6.640	1.124	173.90	17.90	−115.2	−115.9	884	0.01
0.15	−2.19	6.795	1.119	175.18	17.71	−112.4	−116.0	1010	0.05
0.20	−1.67	6.251	1.115	176.26	17.49	−111.3	−117.0	1132	0.09
0.25	−1.36	5.333	1.110	177.15	17.23	−110.9	−118.9	1250	0.13
0.30	−1.17	4.286	1.105	177.86	16.96	−110.8	−122.1	1366	0.19
0.35	−1.05	3.285	1.101	178.42	16.69	−111.0	−127.4	1480	0.28
0.40	−0.96	2.443	1.096	178.84	16.44	−111.5	−135.7	1592	0.40
0.45	−0.90	1.822	1.092	179.13	16.23	−112.0	−147.6	1704	0.56
0.50	−0.87	1.439	1.087	179.31	16.07	−112.4	−161.5	1814	0.73
0.55	−0.84	1.280	1.082	179.40	15.97	−112.3	−172.4	1920	0.83
0.60	−0.82	1.304	1.078	179.41	15.96	−111.6	−175.7	2023	0.82
0.65	−0.82	1.455	1.073	179.36	16.04	−110.7	−173.0	2122	0.71
0.70	−0.82	1.674	1.069	179.26	16.24	−110.0	−168.7	2220	0.59
0.75	−0.82	1.900	1.064	179.14	16.58	−109.4	−165.5	2317	0.48
0.80	−0.83	2.089	1.060	179.00	17.05	−109.0	−164.3	2413	0.39
0.85	−0.84	2.214	1.055	178.86	17.70	−108.6	−165.1	2507	0.30
0.90	−0.85	2.282	1.050	178.75	18.52	−108.3	−167.7	2601	0.21
0.95	−0.87	2.339	1.046	178.66	19.53	−108.0	−170.7	2692	0.12
1.00	−0.89	2.479	1.041	178.63	20.76	−107.6	−171.7	2782	0.00

**Table 9:** Source and purities of the compounds used in this research.

Chemical Name	CAS ${}^{a}$	Source	Purity in Mass Fraction	Analytic Technique ${}^{b}$
Isoniazid	57-83-0	Sigma-Aldrich, Burlington, MA, USA	>0.990	HPLC
Polyethylene glycol 200	25322-68-3	Sigma-Aldrich, Burlington, MA, USA	0.998	GC

${}^{a}$ Chemical Abstracts Service Registry Number. ${}^{b}$ HPLC is high-performance liquid chromatography; GC is gas chromatography.

## Data Availability

Data are contained within the article.

## Abbreviations

The following abbreviations are used in this manuscript:

$C_{P}$	Molar heat capacity
CAS	Chemical Abstracts Service Registry Number
DSC	Differential scanning calorimetry
*e*	Molecular interactions
g	grams
*G*	Gibbs Energy
GC	Gas chromatography
*H*	Enthalpy
HPLC	High-performance liquid chromatography
INH	Isoniazid
id	Ideal
K	Kelvin
kJ	Kilojoule
m	Melting
mix	Mixing
PEG	Polyethylene glycol 200
*R*	Gas constant
*S*	Entropy
sol	Solution
tr	Transfer
*T*	Temperature
$T_{\mathrm{hm}}$	Harmonic temperature
UV	Ultraviolet
W	Water
*w*	Mass fraction
*x*	Mole fraction
γ	Activity coefficient
δ	Solubility parameter
